# Cholera-Infantum

**Published:** 1886-06

**Authors:** 


**Cholera-Infantum.**—Absolute cleanliness is the first thing to be observed. Bathe your child every morning two hours after its breakfast. Keep a flannel bandage about its abdomen throughout the summer. Change all its clothing on putting it to bed at night. Keep it in the open air about eight hours a day. Feed it at regular intervals of about four hours. Offer it pure cold water several times a day. If you feed it artificially, you must pay the strictest attention to the cleanliness of the nursing bottle. You cannot trust this to any one else ; you must attend to it yourself. The bottle when not in use should be be kept standing in cold water. It should also be daily placed in *boiling* water. When a child is actually suffering from this disease give rice water. This has been found highly satisfactory and been the means of saving many a child during such an attack.—*Peoples' Health Journal.*

**Our next number** will contain an article on the subject of Animal Magnetism.

